# 2-Methyl-2-(3-nitro­phen­yl)-1,3-dithiane

**DOI:** 10.1107/S1600536812022283

**Published:** 2012-05-19

**Authors:** Pavla Mirošová, Marek Nečas, Robert Vícha

**Affiliations:** a Department of Chemistry, Faculty of Technology, Tomas Bata University in Zlin, Nám. T. G. Masaryka 275, Zlín 762 72, Czech Republic; b Department of Chemistry, Faculty of Science, Masaryk University, Kamenice 5, Brno-Bohunice 625 00, Czech Republic

## Abstract

The title compound, C_11_H_13_NO_2_S_2_, contains a 1,3-dithiane ring in an almost ideal chair conformation with the following puckering parameters: *Q* = 0.7252 (15) Å, θ = 6.71 (13) and ϕ = 50.4 (11)°. The benzene ring occupies an axial position at the dithiane ring. The nitro group is almost coplanar with the benzene ring [O—N—C—C = −3.2 (2)°]. The mol­ecule has an L-shape with a C—C—C—C torsion angle of −74.15 (17)° for the atoms of the methyl group and the dithiane–benzene linkage. The crystal packing is stabilized only *via* weak non-specific van der Waals inter­actions.

## Related literature

For the preparation of the title compound, see Vícha *et al.* (2011 ▶). For crystallographic data for similar aryl-substituted 1,3-dithia­nes, see: Fun *et al.* (2009*a* ▶,*b* ▶); Samas *et al.* (2010 ▶). For puckering parameters, see: Cremer & Pople (1975 ▶).
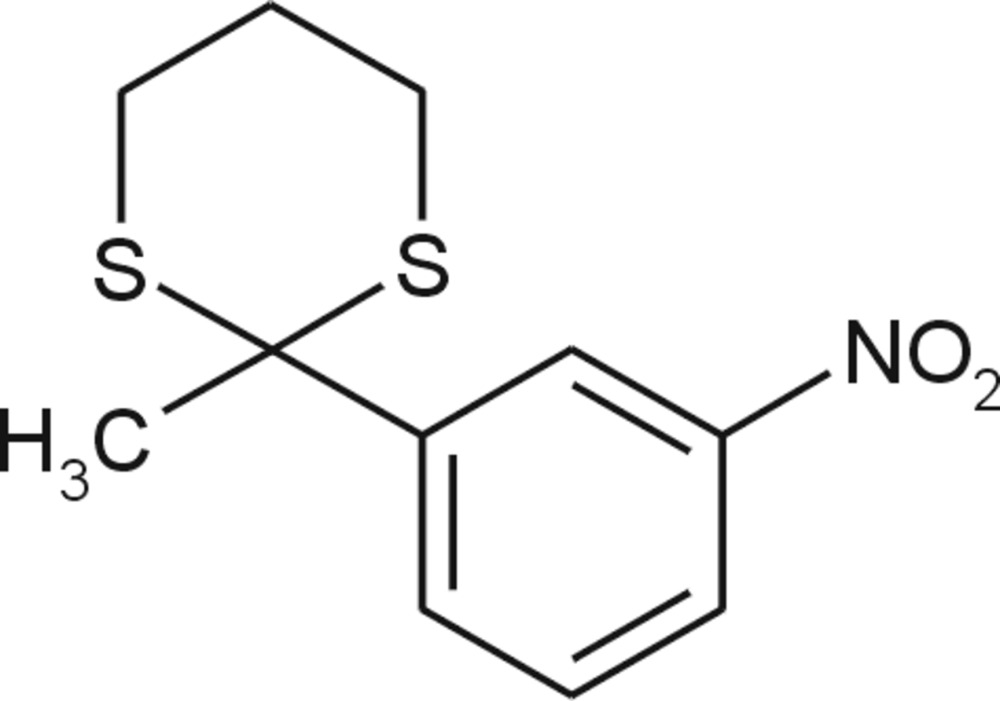


## Experimental

### 

#### Crystal data

C_11_H_13_NO_2_S_2_
*M* *_r_* = 255.36
Orthorhombic, 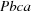

*a* = 13.5388 (3) Å
*b* = 7.2660 (1) Å
*c* = 24.1083 (4) Å
*V* = 2371.60 (7) Å^3^
*Z* = 8
Mo *K*α radiation
μ = 0.43 mm^−1^
*T* = 120 K
0.40 × 0.40 × 0.30 mm

#### Data collection

Oxford Diffraction Xcalibur Sapphire2 diffractometer
Absorption correction: multi-scan (*CrysAlis RED*; Oxford Diffraction, 2009 ▶) *T*_min_ = 0.899, *T*_max_ = 1.000
25370 measured reflections
2086 independent reflections
1871 reflections with *I* > 2σ(*I*)
*R*_int_ = 0.016

#### Refinement

*R*[*F* ^2^ > 2σ(*F* ^2^)] = 0.027
*wR*(*F* ^2^) = 0.074
*S* = 1.08
2086 reflections
145 parameters
H-atom parameters constrained
Δρ_max_ = 0.38 e Å^−3^
Δρ_min_ = −0.19 e Å^−3^

### 

Data collection: *CrysAlis CCD* (Oxford Diffraction, 2009 ▶); cell refinement: *CrysAlis RED* (Oxford Diffraction, 2009 ▶); data reduction: *CrysAlis RED*; program(s) used to solve structure: *SIR92* (Altomare *et al.*, 1993 ▶); program(s) used to refine structure: *SHELXL97* (Sheldrick, 2008 ▶); molecular graphics: *ORTEP-3* (Farrugia, 1997 ▶) and *Mercury* (Macrae *et al.*, 2008 ▶); software used to prepare material for publication: *SHELXL97*.

## Supplementary Material

Crystal structure: contains datablock(s) global, I. DOI: 10.1107/S1600536812022283/nk2160sup1.cif

Structure factors: contains datablock(s) I. DOI: 10.1107/S1600536812022283/nk2160Isup2.hkl

Supplementary material file. DOI: 10.1107/S1600536812022283/nk2160Isup3.cml

Additional supplementary materials:  crystallographic information; 3D view; checkCIF report

## Figures and Tables

**Fig. 1.**
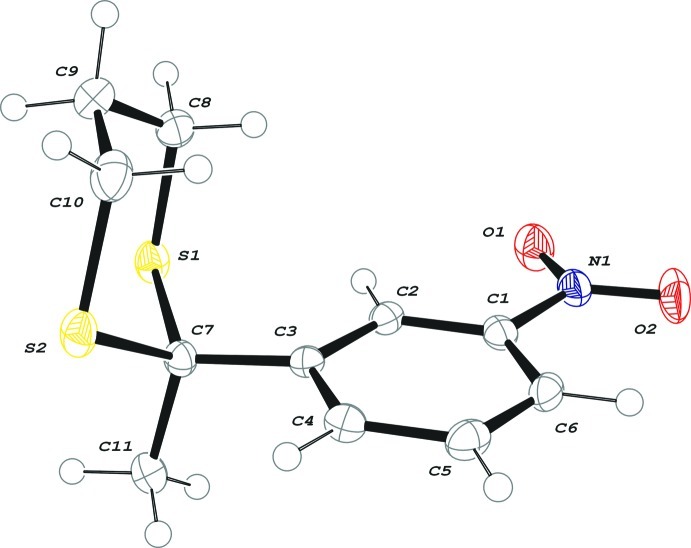
The asymmetric unit with atoms represented as 50% probability ellipsoids. H atoms are shown as small spheres at arbitrary radii.

## supplementary crystallographic information

### Comment

The six- and five-membered 1,3-disulfur rings are frequently used in organic synthesis as efficient protecting groups for carbonyl moiety. Additionally, these compounds are intermediate stage in the carbonyl-to-methylene transformation process. We have used title compound as a model target for optimization of the selective desulfurization procedure (Vícha *et al.*, 2011). Surprisingly, title compound has not been described in the literature yet (to the best of our knowledge).

The benzene ring (C1–C6) is essentially planar with the maximum deviation from the best plane of 0.0071 (15) Å for C4. The torsion angles C11—C7—C3—C4 and C2—C1—N1—O1 describing mutual orientation of nitro group, benzene ring and dithiane ring are -74.15 (17) and -3.2 (2)°, respectively. The dithiane ring adopts almost ideal chair conformation with the Cremer and Pople puckering parameters *Q* = 0.7252 (15) Å, *θ*= 6.71 (13)°, *φ*= 50.4 (11)°. Remarkably, the less bulky methyl substituent occupies the equatorial position at C7. This may be demonstrated by the torsion angle C11—C7—S2—C10 which is of -172.63 (10)°. Furthermore, the C1—C2 edge of the benzene ring is slightly turned over the dithiane ring. The dihedral angle between the benzene best plane (C1–C6) and the imaginary mirror plane of dithiane ring (calculated as the best plane of C3, C7, C9 and C11) is 77.25 (4)°.

### Experimental

The title compound was prepared from corresponding 1-(3-nitrophenyl)ethan-1-one and propan-1,3-dithiol as it was published previously (Vícha *et al.*, 2011). The crude material was crystallized from hexane to yield pale yellow crystals (89%). The single-crystal used for data collection was obtained *via* slow evaporation of chloroform from solution of the title compound at room temperature. NMR, IR and MS spectra are listed in the _exptl_special_details section of the CIF.

### Refinement

All carbon bound H atoms were placed at calculated positions and were refined as riding with their *U*_iso_ set to either 1.2*U*_eq_ or 1.5*U*_eq_ (methyl) of the respective carrier atoms; in addition,the methyl H atoms were allowed to rotate about the C—C bond.

### Crystal data

**Table:**

C_11_H_13_NO_2_S_2_	*D*_x_ = 1.430 Mg m^−^^3^
*M**_r_* = 255.36	Melting point: 350 K
Orthorhombic, *P**b**c**a*	Mo *K*α radiation, λ = 0.71073 Å
Hall symbol: -P 2ac 2ab	Cell parameters from 29781 reflections
*a* = 13.5388 (3) Å	θ = 2.9–27.1°
*b* = 7.2660 (1) Å	µ = 0.43 mm^−^^1^
*c* = 24.1083 (4) Å	*T* = 120 K
*V* = 2371.60 (7) Å^3^	Block, yellow
*Z* = 8	0.40 × 0.40 × 0.30 mm
*F*(000) = 1072	

### Data collection

**Table:**

Oxford Diffraction Xcalibur Sapphire2 diffractometer	2086 independent reflections
Radiation source: Enhance (Mo) X-ray Source	1871 reflections with *I* > 2σ(*I*)
Graphite monochromator	*R*_int_ = 0.016
Detector resolution: 8.4353 pixels mm^-1^	θ_max_ = 25.0°, θ_min_ = 3.3°
ω scan	*h* = −16→10
Absorption correction: multi-scan (*CrysAlis RED*; Oxford Diffraction, 2009)	*k* = −8→8
*T*_min_ = 0.899, *T*_max_ = 1.000	*l* = −28→28
25370 measured reflections	

### Refinement

**Table:**

Refinement on *F*^2^	Primary atom site location: structure-invariant direct methods
Least-squares matrix: full	Secondary atom site location: difference Fourier map
*R*[*F*^2^ > 2σ(*F*^2^)] = 0.027	Hydrogen site location: inferred from neighbouring sites
*wR*(*F*^2^) = 0.074	H-atom parameters constrained
*S* = 1.08	*w* = 1/[σ^2^(*F*_o_^2^) + (0.0353*P*)^2^ + 1.7194*P*] where *P* = (*F*_o_^2^ + 2*F*_c_^2^)/3
2086 reflections	(Δ/σ)_max_ = 0.001
145 parameters	Δρ_max_ = 0.38 e Å^−^^3^
0 restraints	Δρ_min_ = −0.19 e Å^−^^3^

### Special details

**Table:**

Experimental. Spectral properties of title compound: IR (KBr disc): 3075 (w), 2964 (w), 2937 (w), 2897 (w), 2856 (w), 2825 (w),1574 (w), 1524 (*s*), 1468 (w), 1422 (*m*), 1346 (*s*), 1305 (w), 1283 (w), 1274 (w), 1252 (w), 1193 (w), 1166 (w), 1095 (*m*), 1065 (w), 1048 (w), 997 (w), 929 (w), 903 (*m*), 895 (*m*), 870 (w), 805 (*s*), 761 (w), 738 (*s*), 688 (*s*), 626 (*s*), 564 (w), 540 (w), 486 (w) cm^-1^. ^1^H NMR (300 MHz; CDCl_3_): δ 2.75 (s, 3H); 1.94–2.62 (m, 2H); 2.63–2.82 (m, 4H); 7.57 (t, 1H); 8.13–8.16 (m, 1H); 8.31–8.35 (m, 1H); 8.84–8.85 (m, 1H) ppm. ^13^C NMR (75.5 MHz; CDCl_3_): δ 24.5 (CH_2_); 28.3 (CH_2_); 33.0 (CH_3_); 53.3 (C); 122.5 (CH); 123.4 (CH); 129.8 (CH); 134.4 (CH); 147.0 (C);149.0 (C) ppm. MS (EI, 70 eV): 41 (13); 45 (13); 46 (21); 51 (9); 59 (32); 73 (15); 74 (100); 75 (10); 76 (10); 77 (14); 91 (15); 102 (14); 103 (6); 105 (9); 120 (16); 133 (5); 134 (14); 135 (5); 148 (13); 166 (36); 181 (39); 182 (6); 240 (7); 255 (47); 256 (7) m/*z* (%).
Geometry. All e.s.d.'s (except the e.s.d. in the dihedral angle between two l.s. planes) are estimated using the full covariance matrix. The cell e.s.d.'s are taken into account individually in the estimation of e.s.d.'s in distances, angles and torsion angles; correlations between e.s.d.'s in cell parameters are only used when they are defined by crystal symmetry. An approximate (isotropic) treatment of cell e.s.d.'s is used for estimating e.s.d.'s involving l.s. planes.
Refinement. Refinement of *F*^2^ against ALL reflections. The weighted *R*-factor *wR* and goodness of fit *S* are based on *F*^2^, conventional *R*-factors *R* are based on *F*, with *F* set to zero for negative *F*^2^. The threshold expression of *F*^2^ > 2σ(*F*^2^) is used only for calculating *R*-factors(gt) *etc*. and is not relevant to the choice of reflections for refinement. *R*-factors based on *F*^2^ are statistically about twice as large as those based on *F*, and *R*- factors based on ALL data will be even larger.

### Fractional atomic coordinates and isotropic or equivalent isotropic displacement parameters (Å^2^)

**Table:**

	*x*	*y*	*z*	*U*_iso_*/*U*_eq_	
S1	0.42653 (3)	0.06433 (6)	0.318872 (16)	0.02260 (13)	
S2	0.34860 (3)	0.31750 (6)	0.406194 (17)	0.02437 (13)	
O1	0.78844 (9)	0.06238 (18)	0.31090 (5)	0.0316 (3)	
O2	0.88864 (8)	0.19994 (18)	0.36717 (6)	0.0351 (3)	
N1	0.80521 (10)	0.15222 (19)	0.35301 (6)	0.0237 (3)	
C1	0.72158 (11)	0.2054 (2)	0.38863 (6)	0.0193 (3)	
C2	0.62711 (12)	0.1592 (2)	0.37124 (6)	0.0178 (3)	
H2A	0.6174	0.0953	0.3373	0.021*	
C3	0.54695 (11)	0.2075 (2)	0.40399 (6)	0.0169 (3)	
C4	0.56495 (12)	0.3022 (2)	0.45353 (7)	0.0211 (3)	
H4A	0.5108	0.3381	0.4761	0.025*	
C5	0.66055 (13)	0.3445 (2)	0.47029 (7)	0.0241 (4)	
H5A	0.6710	0.4072	0.5044	0.029*	
C6	0.74045 (12)	0.2961 (2)	0.43775 (6)	0.0222 (4)	
H6A	0.8061	0.3244	0.4488	0.027*	
C7	0.44172 (11)	0.1432 (2)	0.39012 (6)	0.0186 (3)	
C8	0.44276 (12)	0.2773 (3)	0.28100 (7)	0.0277 (4)	
H8A	0.5094	0.3267	0.2890	0.033*	
H8B	0.4394	0.2507	0.2408	0.033*	
C9	0.36643 (13)	0.4236 (3)	0.29500 (8)	0.0315 (4)	
H9A	0.3746	0.5290	0.2694	0.038*	
H9B	0.2995	0.3722	0.2892	0.038*	
C10	0.37475 (13)	0.4923 (2)	0.35432 (8)	0.0299 (4)	
H10A	0.3283	0.5961	0.3595	0.036*	
H10B	0.4424	0.5397	0.3604	0.036*	
C11	0.41696 (13)	−0.0232 (2)	0.42694 (7)	0.0265 (4)	
H11A	0.4648	−0.1218	0.4201	0.040*	
H11B	0.3503	−0.0672	0.4182	0.040*	
H11C	0.4199	0.0134	0.4660	0.040*	

### Atomic displacement parameters (Å^2^)

**Table:**

	*U*^11^	*U*^22^	*U*^33^	*U*^12^	*U*^13^	*U*^23^
S1	0.0192 (2)	0.0278 (2)	0.0209 (2)	−0.00284 (16)	−0.00093 (15)	−0.00663 (16)
S2	0.0173 (2)	0.0303 (2)	0.0255 (2)	0.00491 (17)	0.00228 (16)	−0.00487 (17)
O1	0.0243 (7)	0.0412 (7)	0.0293 (6)	−0.0001 (6)	0.0060 (5)	−0.0056 (6)
O2	0.0139 (6)	0.0411 (8)	0.0503 (8)	−0.0024 (5)	−0.0002 (6)	0.0003 (6)
N1	0.0170 (7)	0.0241 (7)	0.0299 (8)	0.0003 (6)	0.0011 (6)	0.0070 (6)
C1	0.0177 (8)	0.0162 (7)	0.0239 (8)	0.0014 (6)	0.0012 (6)	0.0044 (6)
C2	0.0191 (8)	0.0168 (7)	0.0176 (7)	0.0003 (6)	−0.0018 (6)	0.0009 (6)
C3	0.0180 (8)	0.0159 (7)	0.0169 (7)	0.0003 (6)	−0.0009 (6)	0.0028 (6)
C4	0.0233 (8)	0.0196 (8)	0.0204 (8)	0.0002 (7)	0.0016 (6)	0.0004 (6)
C5	0.0300 (9)	0.0212 (8)	0.0210 (8)	−0.0035 (7)	−0.0061 (7)	−0.0007 (6)
C6	0.0200 (8)	0.0192 (8)	0.0275 (8)	−0.0044 (7)	−0.0070 (7)	0.0056 (6)
C7	0.0161 (8)	0.0220 (8)	0.0177 (7)	0.0005 (6)	0.0012 (6)	−0.0018 (6)
C8	0.0229 (9)	0.0414 (10)	0.0189 (8)	−0.0022 (8)	−0.0021 (7)	0.0043 (7)
C9	0.0233 (9)	0.0390 (10)	0.0323 (9)	0.0005 (8)	−0.0048 (8)	0.0118 (8)
C10	0.0227 (9)	0.0245 (9)	0.0426 (10)	0.0038 (7)	−0.0017 (8)	0.0016 (8)
C11	0.0205 (8)	0.0296 (9)	0.0294 (9)	−0.0057 (7)	0.0004 (7)	0.0044 (7)

### Geometric parameters (Å, º)

**Table:**

S1—C8	1.8098 (18)	C5—C6	1.382 (2)
S1—C7	1.8224 (15)	C5—H5A	0.9500
S2—C10	1.8171 (19)	C6—H6A	0.9500
S2—C7	1.8285 (16)	C7—C11	1.537 (2)
O1—N1	1.2282 (18)	C8—C9	1.521 (3)
O2—N1	1.2298 (18)	C8—H8A	0.9900
N1—C1	1.473 (2)	C8—H8B	0.9900
C1—C6	1.379 (2)	C9—C10	1.519 (3)
C1—C2	1.387 (2)	C9—H9A	0.9900
C2—C3	1.387 (2)	C9—H9B	0.9900
C2—H2A	0.9500	C10—H10A	0.9900
C3—C4	1.399 (2)	C10—H10B	0.9900
C3—C7	1.536 (2)	C11—H11A	0.9800
C4—C5	1.390 (2)	C11—H11B	0.9800
C4—H4A	0.9500	C11—H11C	0.9800
			
C8—S1—C7	101.13 (8)	C11—C7—S2	105.78 (11)
C10—S2—C7	101.75 (8)	S1—C7—S2	109.86 (8)
O1—N1—O2	123.30 (14)	C9—C8—S1	113.79 (12)
O1—N1—C1	118.64 (13)	C9—C8—H8A	108.8
O2—N1—C1	118.06 (14)	S1—C8—H8A	108.8
C6—C1—C2	123.08 (15)	C9—C8—H8B	108.8
C6—C1—N1	118.92 (14)	S1—C8—H8B	108.8
C2—C1—N1	117.99 (14)	H8A—C8—H8B	107.7
C1—C2—C3	119.22 (14)	C10—C9—C8	112.84 (14)
C1—C2—H2A	120.4	C10—C9—H9A	109.0
C3—C2—H2A	120.4	C8—C9—H9A	109.0
C2—C3—C4	118.28 (14)	C10—C9—H9B	109.0
C2—C3—C7	121.65 (13)	C8—C9—H9B	109.0
C4—C3—C7	119.78 (14)	H9A—C9—H9B	107.8
C5—C4—C3	121.25 (15)	C9—C10—S2	113.85 (13)
C5—C4—H4A	119.4	C9—C10—H10A	108.8
C3—C4—H4A	119.4	S2—C10—H10A	108.8
C6—C5—C4	120.50 (15)	C9—C10—H10B	108.8
C6—C5—H5A	119.8	S2—C10—H10B	108.8
C4—C5—H5A	119.8	H10A—C10—H10B	107.7
C1—C6—C5	117.65 (15)	C7—C11—H11A	109.5
C1—C6—H6A	121.2	C7—C11—H11B	109.5
C5—C6—H6A	121.2	H11A—C11—H11B	109.5
C3—C7—C11	108.41 (13)	C7—C11—H11C	109.5
C3—C7—S1	113.92 (10)	H11A—C11—H11C	109.5
C11—C7—S1	105.81 (11)	H11B—C11—H11C	109.5
C3—C7—S2	112.50 (11)		
